# The role of short-chain fatty acids in the regulation of osteoporosis: new perspectives from gut microbiota to bone health: A review

**DOI:** 10.1097/MD.0000000000039471

**Published:** 2024-08-23

**Authors:** Boyi Feng, Jingjing Lu, Yanhua Han, Yaguang Han, Xiaokui Qiu, Zhuoying Zeng

**Affiliations:** a Shenzhen Guangming District People’s Hospital, Shenzhen, China; b First Affiliated Hospital, Heilongjiang University of Chinese Medicine, Harbin, China; c Chemical Analysis and Physical Testing Institute, Shenzhen Center for Disease Control and Prevention, Shenzhen, China.

**Keywords:** bone metabolism, gut microbiota, osteoclasts, osteoporosis, short-chain fatty acids.

## Abstract

Osteoporosis is a systemic skeletal disease characterized by low bone density and microarchitectural deterioration, resulting in increased fracture risk. With an aging population, osteoporosis imposes a heavy burden worldwide. Current pharmacotherapies such as bisphosphonates can reduce fracture risk but have limitations. Emerging research suggests that gut microbiota regulates bone metabolism through multiple mechanisms. Short-chain fatty acids (SCFAs) produced from microbial fermentation of dietary fiber beneficially impact bone health. Preclinical studies indicate that SCFAs such as butyrate and propionate prevent bone loss in osteoporosis models by inhibiting osteoclastogenesis and immune modulation. Early clinical data also suggest that SCFA supplementation may improve bone turnover markers in postmenopausal women. SCFAs likely act via inhibition of osteoclast differentiation, stimulation of osteoblast activity, regulation of T cells, and other pathways. However, optimal dosing, delivery methods, and long-term safety require further investigation. Modulating the gut-bone axis via supplementation, prebiotics/probiotics, diet, and lifestyle interventions represents an innovative therapeutic approach for osteoporosis. Harnessing the interplay between microbiome, metabolism, immunity, and bone may provide new directions for managing osteoporosis in the future.

## 1. Introduction

Osteoporosis is one of the most common bone diseases in adults, characterized by a low screening rate, especially among eligible women. Timely treatment of osteoporosis in patients after their first fragility fracture can significantly reduce the risk of subsequent fractures. However, negative reports about bisphosphonate treatments have led to a sharp decline in their use for treating osteoporosis, despite being first-line medications with rare adverse effects.^[1]^ Research indicates that despite the availability of effective treatments, such as calcitonin nasal spray and bisphosphonates, osteoporosis remains widely misunderstood and neglected. Many patients do not receive proper treatment due to a lack of awareness about the disease.^[2]^ Osteoporosis not only impacts individual health but also poses a significant burden on society and the economy. With the aging of the global population, the number of fractures caused by osteoporosis and the associated economic costs are expected to double.

Researchers in a 2023 study discovered a significant link between the gut microbiome and bone density and strength. The study revealed that the abundance of certain bacterial groups negatively correlates with measurements of bone density and microstructure. This suggests that the gut microbiome may indirectly but powerfully regulate bone strength, density, and composition.^[3]^ Some studies assessed the impact of the gut microbiome on bone health by using antibiotics to deplete gut microbes or employing germ-free mouse models. These studies indicate that alterations in the gut microbiome are associated with reduced bone mineral density (BMD). For instance, antibiotic treatment disrupts the composition of the gut microbiome, leading to a decrease in BMD, whereas probiotic treatment with lactobacillus-fermented milk can reverse this effect and promote fracture healing in osteoporotic mice.^[4]^ Various studies underscore the significant impact of the gut microbiome on bone metabolism. The gut microbiome not only affects the bone remodeling process but is also associated with the development of osteoporosis and other bone diseases. These findings highlight the crucial role of the gut microbiome in maintaining bone health and metabolic homeostasis.^[5]^

Short-chain fatty acids (SCFAs) are metabolic byproducts produced by the gut microbiota through the fermentation of indigestible fibers in food, such as dietary fibers. This microbiota primarily includes various anaerobes, such as bifidobacterium and lactobacillus, capable of breaking down cellulose and other complex carbohydrates, resulting in the production of SCFAs such as acetate, propionate, and butyrate. Research has demonstrated that SCFAs can regulate the metabolism of osteoclasts and bone mass, which is of significant importance in the treatment of osteoporosis.^[6]^ Experiments have shown that treatment with SCFAs, such as propionate and butyrate, can significantly increase bone mass and prevent bone loss caused by postmenopausal and inflammatory conditions. This protective effect is associated with the inhibition of osteoclast differentiation and bone resorption while having no impact on bone formation. Propionate and butyrate work by inducing metabolic reprogramming in osteoclasts, enhancing glycolysis, and reducing oxidative phosphorylation, thereby downregulating the expression of osteoclast genes.^[7]^

SCFAs have also been found to exert effects on immune cells, particularly in inducing the generation of regulatory T cells (Tregs). Research suggests that the increased number of Tregs may explain the bone effects of SCFAs, as Tregs have been shown to inhibit osteoclasts and increase bone mass.^[8,9]^ Further research indicates that during the differentiation of osteoclasts, SCFAs such as propionate and butyrate can significantly inhibit key components of bone metabolism signaling, such as TNF receptor-associated factor 6 and nuclear factor of activated T-Cells, cytoplasmic 1. These findings emphasize the direct impact of SCFAs on bone metabolism, particularly in the inhibition of osteoclast differentiation.^[7]^

## 2. Osteoporosis: definition, epidemiology, and treatment

### 2.1. Definition and clinical diagnosis of osteoporosis

Osteoporosis is defined as a systemic skeletal disease characterized by a decrease in bone density and deterioration of bone microarchitecture, leading to an increased risk of fractures. This disease has received extensive attention in the healthcare field over the past 20 years, particularly in terms of screening, diagnosis, and treatment. The diagnosis of osteoporosis is typically based on the measurement of bone mineral density, using the T-score as a key indicator. A T-score of ≤−2.5 is commonly used for the diagnosis of osteoporosis. In addition, depending on various guidelines, the patient’s fracture risk, lifestyle factors (such as intake of calcium and vitamin D, maintaining normal weight, reducing alcohol consumption, and quitting smoking), and pharmacotherapy may also be considered.^[10,11]^ The treatment of osteoporosis includes the use of medications such as bisphosphonates, denosumab, hormone therapy, and parathyroid hormone analogs. Monitoring recommendations for these treatment methods vary, for example, the interval for repeat bone density testing after bisphosphonate therapy differs across major guidelines. Different guidelines also vary in their definition of treatment failure, which is particularly important in the management of osteoporosis.^[12,13]^

### 2.2. Status of epidemiological research on osteoporosis

The global prevalence of osteoporosis demonstrates significant regional variations. A comprehensive systematic review and meta-analysis reported that up to 30% of postmenopausal women, >50% of premenopausal women, and 50% to 80% of men have secondary osteoporosis.^[14]^ Furthermore, with increasing life expectancy and an aging population, osteoporosis is gradually becoming a global epidemic. It is estimated that osteoporosis causes over 8.9 million fractures annually, equivalent to a fracture every 3 seconds. However, it is estimated that only one-third of vertebral fractures due to osteoporosis receive clinical attention.^[15,16]^

### 2.3. Main causes and treatments of osteoporosis

The primary risk factors for osteoporosis include aging, gender (with women at higher risk than men), genetic factors, lifestyle factors (such as dietary habits and physical activity), medication use (especially long-term use of steroids), and other health conditions (such as chronic inflammatory diseases). Recognizing and managing these risk factors are crucial for the prevention of osteoporosis. Current treatment strategies for osteoporosis include pharmacotherapy and lifestyle modifications. Pharmacotherapy mainly comprises bisphosphonates, denosumab, hormone therapy, and parathyroid hormone analogs.^[14]^ Bisphosphonates are commonly used medications for the treatment of osteoporosis, functioning by inhibiting bone resorption to increase bone density and strength. Bisphosphonates can reduce the risk of fractures, particularly in postmenopausal women and older men. Common bisphosphonates include alendronate, risedronate, and zoledronic acid.^[17]^ Recent research has focused on the long-term safety, dosage optimization, and treatment strategies of bisphosphonates. Studies suggest that prolonged use of bisphosphonates may be associated with rare side effects, such as osteonecrosis of the jaw and atypical femoral fractures. Therefore, physicians may recommend regular “drug holidays.” Denosumab, a monoclonal antibody, is used for treating osteoporosis. It reduces bone resorption by inhibiting the action of receptor activator of nuclear factor κB ligand (a bone-resorbing factor), thereby increasing bone density and reducing fracture risk. Denosumab has shown promising results in clinical trials, especially in postmenopausal women. Research has also focused on the long-term effects and safety of denosumab, as well as changes in bone density after discontinuation of the drug.^[18]^ Hormone therapy, particularly estrogen therapy for postmenopausal women, can slow bone loss and increase bone density. However, due to potential increased health risks associated with hormone therapy, such as breast cancer, cardiovascular disease, and thrombosis, it is usually considered only when other treatment options are unsuitable. Current research focuses on assessing the risks and benefits of hormone therapy and determining which patients are most suitable for this type of treatment. Studies are also exploring safer hormone therapy regimens.^[19]^ Parathyroid hormone analogs, such as teriparatide, are medications used for treating osteoporosis that mimic the action of parathyroid hormone, stimulating bone formation, and increasing bone density. Parathyroid hormone analogs have demonstrated the ability to increase bone density and reduce the risk of fractures in clinical trials. Research focuses on the optimal timing, duration of use, and comparison of these drugs with other osteoporosis treatment methods.^[20]^

Overall, these treatment methods are effective in improving bone density and reducing the risk of fractures, but they each have different mechanisms of action, indications, and potential risks. Physicians select the most appropriate treatment plan based on the specific circumstances of the patient. As research progresses, treatment strategies and drug choices are continually evolving and being optimized. In addition to pharmacotherapy, lifestyle adjustments such as supplementing with calcium and vitamin D, maintaining a healthy weight, reducing alcohol consumption, and quitting smoking are also important intervention measures. These treatments aim to reduce the risk of fractures and improve the quality of life for patients.^[21–24]^

## 3. Gut microbiota and bone health

The gut microbiome covers all mucosal surfaces of the host, with the majority residing in the gastrointestinal tract. These microbes collectively promote human health by regulating a variety of biological processes, such as influencing the host’s metabolism and modulating the immune system. Research over the past decade has revealed new connections between the gut microbiome and bone health, particularly in regulating bone metabolism and several metabolic bone diseases, such as osteoporosis, osteoarthritis, and rheumatoid arthritis.^[25–27]^

The gut microbiome influences bone health in multiple ways. It aids in the absorption of nutrients and maintains the integrity of the intestinal barrier, thereby enhancing BMD. In addition, the microbiome regulates several key hormones involved in bone metabolism, such as estrogen, testosterone, insulin-like growth factor-1, parathyroid hormone, serotonin, and gastrointestinal hormones, thereby impacting bone health. For example, estrogen plays a crucial role in maintaining bone health by reducing bone resorption through the maintenance of systemic and bone marrow T cell homeostasis and also directly regulates the formation of osteoblasts and osteoclasts.^[28–30]^

Recent studies highlight the significant impact of gut microbiome composition on bone health. For instance, the removal of gut microbiota through antibiotic treatment has been observed to improve bone mass, microarchitecture, and strength in ovariectomized mice. Conversely, transplanting gut microbiota adapted to ovariectomy leads to bone loss. However, gene deletion reversed the tibial gene expression induced by ovariectomy, increasing periosteal bone formation.^[31]^

Given that dysbiosis of the gut microbiome is associated with various bone diseases, modulating the composition of the gut microbiome may be an effective strategy for preventing and treating these conditions. For example, probiotics, prebiotics, and fecal microbiota transplantations are preferred methods for restoring a disturbed gut microbiome. Moreover, metabolic products produced by the gut microbiome, particularly SCFAs and bile acids, also play a role in bone homeostasis and hold promise as effective strategies for clinically treating gut microbiome-related disorders in the future. These research findings indicate that the gut microbiome plays a key role in maintaining bone metabolism through its interactions with multiple physiological systems.^[32–34]^

In summary, the gut microbiome significantly impacts bone health, participating in the regulation of bone metabolism through various mechanisms. The composition and activity of the gut microbiome are closely linked to the development of bone diseases; hence, modulating the gut microbiome may become an effective strategy for treating these conditions. However, research in this field is still evolving, and more clinical trials are needed in the future to ascertain the efficacy of these findings in humans.

## 4. Effects of SCFAs on osteoporosis

### 4.1. Classification and biochemical characteristics of SCFAs

SCFAs, primarily acetate (C2), propionate (C3), and butyrate (C4), are fatty acids with fewer than 6 carbon atoms. They are the end products of the fermentation of dietary fibers by the gut microbiota. These SCFAs have distinct biochemical properties: acetate (C2), the most abundant SCFA in the colon. It plays a crucial role in cholesterol metabolism and lipogenesis. Propionate (C3): it is primarily absorbed by the liver and is known for its role in gluconeogenesis and regulation of fatty acid synthesis. Butyrate (C4): a critical energy source for colonic epithelial cells; butyrate also possesses anti-inflammatory properties and plays a role in cell differentiation and apoptosis.^[35,36]^

### 4.2. SCFA production, absorption, and metabolic pathways

Production: SCFAs are produced through the microbial fermentation of indigestible dietary fibers in the colon. The composition of the gut microbiota determines the types and amounts of SCFAs produced. Absorption: SCFAs are readily absorbed by the cells lining the colon and are then transported to the liver via the portal vein. Metabolic pathways: once absorbed, SCFAs can be utilized as energy sources or incorporated into various metabolic pathways. Acetate circulates throughout the body and can be utilized peripherally, while propionate is largely taken up by the liver.^[37–39]^

### 4.3. SCFA receptors and signal transduction mechanisms

Receptors: SCFAs exert their effects via several mechanisms, including the activation of G protein-coupled receptors such as GPR41 (free fatty acid receptor 3), GPR43 (free fatty acid receptor 2), and GPR109A. Signal transduction: these receptors trigger a variety of intracellular signaling pathways, influencing processes such as hormone secretion, inflammation modulation, and immune cell function.^[40–42]^

### 4.4. Effects of SCFAs on intestinal barrier, immune system, and hormone levels

Intestinal barrier: SCFAs strengthen the intestinal barrier by promoting the integrity and function of the epithelial cells. Butyrate, in particular, enhances the production of tight junction proteins.^[42]^ Immune system: SCFAs have a profound impact on the immune system. They modulate the function of immune cells, including T cells and macrophages, and play a role in reducing inflammation.^[8,9,28,30]^ Hormone levels: SCFAs influence the secretion of various gut hormones, such as glucagon-like peptide-1, which can impact appetite regulation and glucose homeostasis.^[43–45]^ The understanding of SCFAs has greatly expanded, revealing their multifaceted roles in human health and disease. Continued research in this area is likely to uncover more about their potential therapeutic applications, especially in the context of metabolic diseases and conditions such as osteoporosis.

## 5. SCFAs and bone health

### 5.1. Effects of SCFAs on bone density and quality

Bone density improvement: studies have shown that SCFAs, particularly butyrate and propionate, can enhance bone density. They do this by influencing the activity of osteoblasts (bone-forming cells) and osteoclasts (bone-resorbing cells), thus promoting bone formation and reducing bone resorption.^[46]^

Bone quality enhancement: SCFAs contribute to the improvement of bone quality by influencing the mineralization process and the bone matrix’s quality. They may alter the composition of the bone, making it more resistant to fractures.^[7]^

### 5.2. Effects of SCFAs on bone metabolism-related cells

Osteoclasts: SCFAs, especially butyrate, have been found to inhibit the formation and function of osteoclasts. This effect is crucial for preventing excessive bone resorption, which is a hallmark of osteoporosis.^[7,46]^

Osteoblasts: SCFAs can stimulate the proliferation and differentiation of osteoblasts. This is particularly important for the maintenance and repair of bone tissue. The mechanism might involve the activation of specific signaling pathways that promote osteogenic activity.^[47,48]^

### 5.3. Role of SCFAs in regulating bone marrow stromal cells and bone metabolic factors

Influence on bone marrow stromal cells: SCFAs have been shown to impact the function of bone marrow stromal cells, which are progenitor cells capable of differentiating into osteoblasts. SCFAs may influence the fate of these cells, tilting them toward bone-forming activities. Regulation of bone metabolic factors: SCFAs can modulate the expression of various factors involved in bone metabolism. This includes the regulation of cytokines, growth factors, and hormones that are critical for maintaining bone homeostasis. For instance, they can modulate the levels of receptor activator of nuclear factor κB ligand and osteoprotegerin, 2 critical regulators of osteoclastogenesis.^[49,50]^

In summary, SCFAs play a significant role in bone health by affecting bone density, quality, and the activity of cells involved in bone metabolism. Their influence extends from the cellular level, affecting osteoblasts and osteoclasts, to the systemic level, where they impact the overall bone metabolic environment. Understanding these mechanisms is crucial for developing new therapeutic strategies for bone-related diseases such as osteoporosis.

## 6. SCFAs in the treatment of osteoporosis

### 6.1. Exploring the potential of SCFAs as a treatment for osteoporosis

Mechanism of action: discuss how SCFAs influence bone metabolism, focusing on cellular and molecular pathways. Preclinical studies: summarize animal studies that have explored the effects of SCFAs on bone density and strength. Human research: highlight any clinical trials or observational studies in humans that indicate the potential benefits of SCFAs in preventing or treating osteoporosis.^[51,52]^

### 6.2. Research progress of SCFA supplements and related receptor agonists/antagonists

Development of supplements: review the current status of SCFA supplements, including their composition, bioavailability, and safety. Receptor targets: detail the research on specific SCFA receptors such as GPR41, GPR43, and their role in bone health.

Advances in agonists/antagonists: cover the latest developments in creating or identifying receptor agonists/antagonists that could be used in osteoporosis therapy.^[53,54]^

## 7. Conclusion and future prospect

### 7.1. Summarizing the potential role of SCFAs in osteoporosis prevention and treatment

In summary, emerging evidence suggests that SCFAs play an important role in bone health and have potential as a novel strategy for osteoporosis prevention and treatment. Preclinical studies have demonstrated that SCFAs, especially butyrate and propionate, can prevent bone loss and improve bone strength in animal models of osteoporosis. The mechanisms likely involve inhibition of osteoclast differentiation, stimulation of osteoblast activity, and immunomodulatory effects through regulation of T cells. Early clinical trials also indicate the beneficial effects of SCFA supplementation on biomarkers of bone metabolism in postmenopausal women.

Overall, SCFAs show promise as a supplemental approach for reducing bone loss and fracture risk in patients with osteoporosis. SCFAs could potentially be used as a safe adjunct to standard pharmacotherapy. However, larger randomized controlled trials are needed to definitively establish clinical efficacy. Developing targeted delivery methods and optimizing the composition of SCFA supplements will also be important areas of future research.

### 7.2. Limitations and future research directions

While preliminary findings are encouraging, there are several limitations to current research. Most studies have been conducted in animal models, with few clinical trials in humans so far. The optimal dosing, formulation, and duration of SCFA treatment need to be determined. Effects may depend on the specific composition of the SCFA mixture. Long-term studies are required to ascertain the safety of chronic SCFA administration. Interactions with medications for osteoporosis also need investigation.

In addition, the mechanisms of action are not yet fully elucidated. More research is needed on the molecular pathways and immune cells involved in the bone effects of SCFAs. Differences between individual SCFAs such as butyrate and propionate should be clarified. Exploring the bone-protective effects of SCFA receptor agonists is another important area for future work. Multipronged approaches examining SCFAs, gut microbiome, diet, and lifestyle factors may provide further insights.

### 7.3. Outlook on innovative ideas for osteoporosis treatment strategies

The gut-bone axis is an exciting new frontier in osteoporosis research. SCFAs are likely one piece of this complex interplay between the microbiome, metabolism, immune function, and bone. Harnessing this knowledge could lead to novel treatment paradigms that go beyond traditional pharmacotherapy.

Future osteoporosis management may involve an integrated approach including SCFA supplementation, probiotics/prebiotics to favor bone-protective microbial profiles, anti-inflammatory diets high in fiber, and lifestyle measures to optimize nutrition and exercise. Personalized strategies based on an individual’s microbiome and metabotype could be developed. Advances in delivery methods, such as nanoparticles to target SCFAs to bone, may enhance efficacy. More research on microbiome-based diagnostics could also help refine fracture risk assessments and therapeutic decisions.

In conclusion, modulation of the gut-bone axis via SCFAs and related approaches represents an exciting new frontier. While still early, the translation of these scientific insights into clinical practice could transform osteoporosis care in the future. More rigorous research and innovation are needed to realize this potential.

## Author contributions

**Formal analysis:** Boyi Feng, Jingjing Lu.

**Methodology:** Boyi Feng.

**Writing – original draft:** Boyi Feng.

**Writing – review & editing:** Jingjing Lu, Yanhua Han, Yaguang Han, Xiaokui Qiu, Zhuoying Zeng.

**Funding acquisition:** Zhuoying Zeng.

**Project administration:** Zhuoying Zeng.

## References

[1] Gopinath V. Osteoporosis. Med Clin North Am. 2023;107:213–25. 36759092 10.1016/j.mcna.2022.10.013

[2] Foessl I Dimai HP Obermayer-Pietsch B. Long-term and sequential treatment for osteoporosis. Nat Rev Endocrinol. 2023;19:520–33. 37464088 10.1038/s41574-023-00866-9

[3] Lahiri S Kim H Garcia-Perez I. The gut microbiota influences skeletal muscle mass and function in mice. Sci Transl Med. 2019;11:eaan5662. 31341063 10.1126/scitranslmed.aan5662 PMC7501733

[4] Srivastava RK Duggal NA Parameswaran N. Editorial: gut microbiota and gut-associated metabolites in bone health. Front Endocrinol (Lausanne). 2023;14:1232050. 37547322 10.3389/fendo.2023.1232050 PMC10400440

[5] Tu Y Yang R Xu X Zhou X. The microbiota-gut-bone axis and bone health. J Leukoc Biol. 2021;110:525–37. 33884666 10.1002/JLB.3MR0321-755R

[6] Srivastava RK Sapra L Mishra PK. Osteometabolism: metabolic alterations in bone pathologies. Cells. 2022;11:3943. 36497201 10.3390/cells11233943 PMC9735555

[7] Lucas S Omata Y Hofmann J. Short-chain fatty acids regulate systemic bone mass and protect from pathological bone loss. Nat Commun. 2018;9:55. 29302038 10.1038/s41467-017-02490-4 PMC5754356

[8] Parada Venegas D De la Fuente MK Landskron G. Short chain fatty acids (SCFAs)-mediated gut epithelial and immune regulation and its relevance for inflammatory bowel diseases. Front Immunol. 2019;10:277. 30915065 10.3389/fimmu.2019.00277 PMC6421268

[9] Yang W Yu T Huang X. Intestinal microbiota-derived short-chain fatty acids regulation of immune cell IL-22 production and gut immunity. Nat Commun. 2020;11:4457. 32901017 10.1038/s41467-020-18262-6 PMC7478978

[10] Lorentzon M Cummings SR. Osteoporosis: the evolution of a diagnosis. J Intern Med. 2015;277:650–61. 25832448 10.1111/joim.12369

[11] Reid IR. A broader strategy for osteoporosis interventions. Nat Rev Endocrinol. 2020;16:333–9. 32203407 10.1038/s41574-020-0339-7

[12] Harris K Zagar CA Lawrence KV. Osteoporosis: common questions and answers. Am Fam Physician. 2023;107:238–46. 36920813

[13] Black DM Rosen CJ. Clinical practice. Postmenopausal osteoporosis. N Engl J Med. 2016;374:254–62. 26789873 10.1056/NEJMcp1513724

[14] Ebeling PR Nguyen HH Aleksova J Vincent AJ Wong P Milat F. Secondary osteoporosis. Endocr Rev. 2022;43:240–313. 34476488 10.1210/endrev/bnab028

[15] Salari N Darvishi N Bartina Y. Global prevalence of osteoporosis among the world older adults: a comprehensive systematic review and meta-analysis. J Orthop Surg Res. 2021;16:669. 34774085 10.1186/s13018-021-02821-8 PMC8590304

[16] Salari N Ghasemi H Mohammadi L. The global prevalence of osteoporosis in the world: a comprehensive systematic review and meta-analysis. J Orthop Surg Res. 2021;16:609. 34657598 10.1186/s13018-021-02772-0 PMC8522202

[17] Tutaworn T Nieves JW Wang Z Levin JE Yoo JE Lane JM. Bone loss after denosumab discontinuation is prevented by alendronate and zoledronic acid but not risedronate: a retrospective study. Osteoporos Int. 2023;34:573–84. 36602607 10.1007/s00198-022-06648-9 PMC9813893

[18] Reid IR Billington EO. Drug therapy for osteoporosis in older adults. Lancet. 2022;399:1080–92. 35279261 10.1016/S0140-6736(21)02646-5

[19] Ayers C Kansagara D Lazur B Fu R Kwon A Harrod C. Effectiveness and safety of treatments to prevent fractures in people with low bone mass or primary osteoporosis: a living systematic review and network meta-analysis for the American College of Physicians. Ann Intern Med. 2023;176:182–95. 36592455 10.7326/M22-0684

[20] Lorentzon M. Treating osteoporosis to prevent fractures: current concepts and future developments. J Intern Med. 2019;285:381–94. 30657216 10.1111/joim.12873

[21] Mendez-Sanchez L Clark P Winzenberg TM Tugwell P Correa-Burrows P Costello R. Calcium and vitamin D for increasing bone mineral density in premenopausal women. Cochrane Database Syst Rev. 2023;1:CD012664. 36705288 10.1002/14651858.CD012664.pub2 PMC9881395

[22] Liu C Kuang X Li K Guo X Deng Q Li D. Effects of combined calcium and vitamin D supplementation on osteoporosis in postmenopausal women: a systematic review and meta-analysis of randomized controlled trials. Food Funct. 2020;11:10817–27. 33237064 10.1039/d0fo00787k

[23] Reis AR Santos RKF Dos Santos CB. Supplementation of vitamin D isolated or calcium-associated with bone remodeling and fracture risk in postmenopausal women without osteoporosis: a systematic review of randomized clinical trials. Nutrition. 2023;116:112151. 37544189 10.1016/j.nut.2023.112151

[24] Capozzi A Scambia G Lello S. Calcium, vitamin D, vitamin K2, and magnesium supplementation and skeletal health. Maturitas. 2020;140:55–63. 32972636 10.1016/j.maturitas.2020.05.020

[25] Tu Y Kuang X Zhang L Xu X. The associations of gut microbiota, endocrine system and bone metabolism. Front Microbiol. 2023;14:1124945. 37089533 10.3389/fmicb.2023.1124945 PMC10116073

[26] Lu L Chen X Liu Y Yu X. Gut microbiota and bone metabolism. FASEB J. 2021;35:e21740. 34143911 10.1096/fj.202100451R

[27] D’Amelio P Sassi F. Gut microbiota, immune system, and bone. Calcif Tissue Int. 2018;102:415–25. 28965190 10.1007/s00223-017-0331-y

[28] Yang X Zhou F Yuan P. T cell-depleting nanoparticles ameliorate bone loss by reducing activated T cells and regulating the Treg/Th17 balance. Bioact Mater. 2021;6:3150–63. 33778195 10.1016/j.bioactmat.2021.02.034 PMC7970013

[29] Guo M Liu H Yu Y. Lactobacillus rhamnosus GG ameliorates osteoporosis in ovariectomized rats by regulating the Th17/Treg balance and gut microbiota structure. Gut Microbes. 2023;15:2190304. 36941563 10.1080/19490976.2023.2190304 PMC10038048

[30] Lorenzo J. From the gut to bone: connecting the gut microbiota with Th17 T lymphocytes and postmenopausal osteoporosis. J Clin Invest. 2021;131:e146619. 33645543 10.1172/JCI146619 PMC7919711

[31] Guan Z Xuanqi Z Zhu J. Estrogen deficiency induces bone loss through the gut microbiota. Pharmacol Res. 2023;196:106930. 37722518 10.1016/j.phrs.2023.106930

[32] Zhang YW Cao MM Li YJ. The regulative effect and repercussion of probiotics and prebiotics on osteoporosis: involvement of brain-gut-bone axis. Crit Rev Food Sci Nutr. 2023;63:7510–28. 35234534 10.1080/10408398.2022.2047005

[33] Li JY Chassaing B Tyagi AM. Sex steroid deficiency-associated bone loss is microbiota dependent and prevented by probiotics. J Clin Invest. 2016;126:2049–63. 27111232 10.1172/JCI86062 PMC4887186

[34] Barrea L Verde L Auriemma RS. Probiotics and prebiotics: any role in menopause-related diseases? Curr Nutr Rep. 2023;12:83–97. 36746877 10.1007/s13668-023-00462-3 PMC9974675

[35] Wong JM de Souza R Kendall CW Emam A Jenkins DJ. Colonic health: fermentation and short chain fatty acids. J Clin Gastroenterol. 2006;40:235–43. 16633129 10.1097/00004836-200603000-00015

[36] van de Wouw M Boehme M Lyte JM. Short-chain fatty acids: microbial metabolites that alleviate stress-induced brain-gut axis alterations. J Physiol. 2018;596:4923–44. 30066368 10.1113/JP276431 PMC6187046

[37] Ramos Meyers G Samouda H Bohn T. Short chain fatty acid metabolism in relation to gut microbiota and genetic variability. Nutrients. 2022;14:5361. 36558520 10.3390/nu14245361 PMC9788597

[38] Zietek M Celewicz Z Szczuko M. Short-chain fatty acids, maternal microbiota and metabolism in pregnancy. Nutrients. 2021;13:1244. 33918804 10.3390/nu13041244 PMC8069164

[39] Ikeda T Nishida A Yamano M Kimura I. Short-chain fatty acid receptors and gut microbiota as therapeutic targets in metabolic, immune, and neurological diseases. Pharmacol Ther. 2022;239:108273. 36057320 10.1016/j.pharmthera.2022.108273

[40] Li YJ Chen X Kwan TK. Dietary fiber protects against diabetic nephropathy through short-chain fatty acid-mediated activation of G protein-coupled receptors GPR43 and GPR109A. J Am Soc Nephrol. 2020;31:1267–81. 32358041 10.1681/ASN.2019101029 PMC7269358

[41] Kimura I Ichimura A Ohue-Kitano R Igarashi M. Free fatty acid receptors in health and disease. Physiol Rev. 2020;100:171–210. 31487233 10.1152/physrev.00041.2018

[42] de Vos WM Tilg H Van Hul M Cani PD. Gut microbiome and health: mechanistic insights. Gut. 2022;71:1020–32. 35105664 10.1136/gutjnl-2021-326789 PMC8995832

[43] Psichas A Sleeth ML Murphy KG. The short chain fatty acid propionate stimulates GLP-1 and PYY secretion via free fatty acid receptor 2 in rodents. Int J Obes (Lond). 2015;39:424–9. 25109781 10.1038/ijo.2014.153 PMC4356745

[44] Ma Y Lee E Yoshikawa H. Phloretin suppresses carbohydrate-induced GLP-1 secretion via inhibiting short chain fatty acid release from gut microbiome. Biochem Biophys Res Commun. 2022;621:176–82. 35841764 10.1016/j.bbrc.2022.06.069

[45] Min Q Wang Y Jin T. Analysis of intestinal short-chain fatty acid metabolism profile after probiotics and GLP-1 treatment for type 2 diabetes mellitus. Front Endocrinol (Lausanne). 2022;13:892127. 35846273 10.3389/fendo.2022.892127 PMC9280620

[46] Fan D Miao J Fan X Wang Q Sun M. Effects of valproic acid on bone mineral density and bone metabolism: a meta-analysis. Seizure. 2019;73:56–63. 31756600 10.1016/j.seizure.2019.10.017

[47] Kondo T Chiba T Tousen Y. Short-chain fatty acids, acetate and propionate, directly upregulate osteoblastic differentiation. Int J Food Sci Nutr. 2022;73:800–8. 35616294 10.1080/09637486.2022.2078285

[48] Wallimann A Magrath W Thompson K. Gut microbial-derived short-chain fatty acids and bone: a potential role in fracture healing. Eur Cell Mater. 2021;41:454–70. 33881768 10.22203/eCM.v041a29 PMC9100835

[49] Chang MC Chen YJ Lian YC. Butyrate stimulates histone H3 acetylation, 8-isoprostane production, RANKL expression, and regulated osteoprotegerin expression/secretion in MG-63 osteoblastic cells. Int J Mol Sci . 2018;19:4071. 30562925 10.3390/ijms19124071 PMC6321057

[50] Song W Sheng Q Bai Y. Obesity, but not high-fat diet, is associated with bone loss that is reversed via CD4^+^CD25^+^Foxp3^+^ Tregs-mediated gut microbiome of non-obese mice. NPJ Sci Food. 2023;7:14. 37055440 10.1038/s41538-023-00190-6 PMC10102288

[51] Lecomte M Tomassi D Rizzoli R. Effect of a hop extract standardized in 8-prenylnaringenin on bone health and gut microbiome in postmenopausal women with osteopenia: a one-year randomized, double-blind, placebo-controlled trial. Nutrients. 2023;15:2688. 37375599 10.3390/nu15122688 PMC10304064

[52] Zhao F Guo Z Kwok LY. Bifidobacterium lactis Probio-M8 improves bone metabolism in patients with postmenopausal osteoporosis, possibly by modulating the gut microbiota. Eur J Nutr. 2023;62:965–76. 36334119 10.1007/s00394-022-03042-3

[53] Kim MH Kang SG Park JH Yanagisawa M Kim CH. Short-chain fatty acids activate GPR41 and GPR43 on intestinal epithelial cells to promote inflammatory responses in mice. Gastroenterology. 2013;145:396–406.e1. 23665276 10.1053/j.gastro.2013.04.056

[54] Sivaprakasam S Prasad PD Singh N. Benefits of short-chain fatty acids and their receptors in inflammation and carcinogenesis. Pharmacol Ther. 2016;164:144–51. 27113407 10.1016/j.pharmthera.2016.04.007 PMC4942363

